# Comparative evaluation of QuEChERS and the ‘dilute and shoot’ QuPPe extraction methods coupled with LC-MS/MS for the analysis of mepiquat residue in sweet potatoes: addressing residual soil impact on recovery efficiency

**DOI:** 10.1038/s41598-026-37007-x

**Published:** 2026-02-12

**Authors:** Mohamed Wageed, Hend A. Mahmoud, M. I. Abdel-Megeed, Ramadan Sayed, Hassan A. El-Gammal, Mostafa Soliman

**Affiliations:** 1 Agricultural Research Center, Central Laboratory of residue Analysis of Pesticides and Heavy Metals in Foods (QCAP), Ministry of Agriculture and Land Reclamation, 7 Nadi El Said St., Dokki, Giza, 12311 Egypt; 2 Plant Protection Department, Faculty of Agriculture, Ain Shams University, Cairo, Egypt; 3 Department of Chemistry, Faculty of Science, Al-Azhar University, Nasr City, Cairo Egypt

**Keywords:** Mepiquat, QuPPe, QuEChERS, Sweet potatoes, LC-MS/MS, Soil, Ecology, Ecology, Environmental sciences, Plant sciences

## Abstract

Mepiquat (MPQ) is a quaternary ammonium salt widely used as a growth regulator in agriculture. A recent report from the European Union Reference Laboratory for Single Residue Methods (EURL SRM) reported MPQ residues in Egyptian sweet potatoes at levels that raised some concern. Hence, this study aimed to develop and validate a fast, effective extraction method coupled with LC-MS/MS for MPQ analysis in Egypt. Initially, the QuEChERS citrate buffered method was tested, but it showed significant fluctuation in spike recovery (30%-72%). A systematic investigation revealed that recovery differed depending on the amount of residual soil in the sweet potato samples. Two key observations were made: (1) Clay loam negatively affected recovery more than sandy loam, and (2) the “dilute and shoot” Quick Polar Pesticides (QuPPe) method was less impacted by residual soil. By applying exponential decay equation modelling, it was proved that residual soil can remain on the sweet potato even after 10 min washing. A study on the effect of washing for 10 min was done using real contaminated samples and it showed that about 30% of the incurred pesticide was lost. Hence, dry scrubbing was used for the removal of the soil on the sweet potato, in addition to the QuPPe method for sample extraction. This provides a predictive framework for understanding how residual soil influences recovery, which could be extended to other polar pesticides and root crops. The QuPPe method was validated for specificity, selectivity, accuracy, and precision, ensuring its suitability for quantitative MPQ analysis in sweet potatoes. A survey of 30 samples showed that only four tested positives for MPQ, with one exceeding the maximum residue limits.

## Introduction

Mepiquat (MPQ), is the common name for the quaternary ammonium salt (QUATs) 1,1 dimethylpiperidinium cation, a broadly applied plant growth regulator in agriculture, usually in the chlorinated form^1–3^. MPQ is applied on numerous food crops such as cereals, fruits and vegetables to control excessive shoot growth without affecting plant productivity^4–6^. MPQ is characterized by high water solubility (log Kow = -3.55 at 20 °C and pH 7) ^3,7^ non-volatility, and thermal stability in acidic environments^8^.

Sweet potatoes (Ipomoea batatas L.), an essential tropical tuber crop that belongs to the Convolvulaceae family^9^. It ranks as the seventh position in global food crop importance, coming after wheat, rice, corn, potatoes, barley, and cassava^10,11^. Sweet potatoes are rich in carbohydrates, sugary and it’s regarded as a valuable source for vitamin C, iron, provitamin A, and other important minerals^12,13^. In 2023, sweet potatoes were grown on approximately 12,427 hectares in Egypt, yielding around 423,423.19 tons. In 2021, Egyptian sweet potatoes’ exports reached about 184,000 tons^14^. Making sweet potatoes an essential crop in both local market and exported commodities for Egypt.

Pesticide residues are an essential criterion in permitting agricultural products to enter importing countries and parties such as the European Union (EU) countries^15^. The maximum residue limit (MRL) is defined as the highest permitted concentration of pesticide residue on food or feed when applied in accordance with Good Agricultural Practices^16^. The EU usually establishes a default MRL for unexamined substances equals to the Limit of quantification (LOQ)^17^. For example, the EU set an MRL value of 0.02 mg/kg for MPQ in sweet potatoes while the MRL is 40 mg/kg in Linseeds^18^.

An alarming report from the European Union Reference Laboratory for Pesticides Requiring Single Residue Methods (EURL-SRM) reported the presence of MPQ residue equals to 0.04 mg/kg in sweet potatoes in 2023 ^19^ which raised concerns and triggered more rigorous monitoring and control on the residues of MPQ in sweet potatoes that are imported from Egypt. Accordingly, the presence of an appropriate analytical method for monitoring and controlling MPQ residue in necessary to facilitate Egyptian exports of sweet potatoes.

Globally, the quick, easy, cheap, effective, rigid, and safe (QuEChERS), is the most widely used sample preparation method for the determination of pesticides in food^20^. A quick search using “QuEChERS” as keyword on Scopus search engine yielded 3165 results. The EU reference method for multi residue determination of pesticides is the citrate buffered QuEChERS number EN 15,662. However, some pesticides require special treatment and can’t be extracted using QuEChERS. MPQ is an example due to its high polarity. Hence, the EURL-SRM has developed another “dilute and shoot” method named the Quick Polar Pesticides (QuPPe) method for the determination of polar pesticides (log *K*ow values < -1) ^21^ such as MPQ. However, due to its borderline polarity, QuEChERS can be used for the determination of MPQ in some commodities. For example, Urban et al. used a modified QuEChERS version to analyze MPQ in oats and whole wheat grain^8^. Also, Gao et al. applied the method to detect it in potatoes and pears^6^.

The use of QuEChERS in the determination of MPQ can be very efficient in terms of time and cost as QuEChERS can be used as a sample preparation method for the co-determination of up to 400 pesticides^22–24^. On the contrary, QuPPe is specific for only highly polar pesticides. Hence, this study aimed to assess the efficiency of QuEChERS technique in comparison to the QuPPe method for the determination of MPQ in sweet potatoes by applying liquid chromatography and tandem mass spectrometry (LC-MS/MS). The optimum method was validated according to SANTE/11,312/2021v2 guidelines^25^. The validated method was utilized to monitor MPQ in thirty sweet potatoe samples collected from the Egyptian market.

## Materials and methods

### Chemicals and reagents

The MPQ reference standard (98% purity) was obtained from Dr. Ehrenstorfer (Germany). Methanol and acetonitrile, both LC-MS grade, were acquired from Merck (Germany). Formic acid (90% purity) was supplied by John Townsend Baker (USA). Deionized water was prepared using a Millipore Integral System from Merck (Germany). QuEChERS citrate buffer based extraction kits were purchased from Agilent (USA). Ammonium acetate (98% purity) was sourced from Sigma-Aldrich (USA).

### Standard preparation

A stock solution of MPQ was prepared at a concentration of 1000 µg/mL by dissolving the reference standard in methanol. Key properties of MPQ are summarized in Table 1. From this stock solution, a 10 µg/mL working standard of MPQ was prepared. Calibration standards were then prepared in acetonitrile at concentrations ranging from 0.005 to 0.5 µg/mL. All prepared solutions were stored at − 20 °C to ensure stability.

**Table 1:** MPQ key information.

Pesticide name	Mepiquat
Molecular structure	
IUPAC name	1,1-dimethylpiperidin-1-ium
Molecular Weight	114.21 g/mol
Molecular formula	C7H16N+
CAS-Number	15302-91-7
Mode of action	plant growth inhibitors
Octanol-water partition coefficient (Log POW)	pH 7, 20 ◦C= -3.55

### HPLC–MS/MS instrumentation and condition

The analysis was conducted using an AB SCIEX Triple Quad 6500 + LC-MS/MS system fitted with an IonDrive Turbo V source, operating in positive Electrospray Ionization (ESI) mode. The system was coupled with a Shimadzu HPLC system (Exion LC) supplied by AB SCIEX (Germany). Chromatographic separation of MPQ was achieved using an Agilent PoroShell 120 EC-C18 column (2.7 μm, 3.3 mm × 50 mm) from Agilent (USA), with the column temperature maintained at 40 °C. The mobile phase comprised two components: solvent A (10 mM ammonium acetate buffer, pH 4) and solvent B (acetonitrile). An isocratic elution program (10% A: 90% B) was employed at a flow rate of 0.3 mL/min for a 5-minute run.

The mass spectrometric conditions were optimized as follows: ion spray voltage of 5.5 kV, ion source temperature of 450 °C, curtain gas pressure of 25 psi, atomization air pressure of 40 psi, auxiliary gas pressure of 45 psi, and entrance potential of 10 V. Detection and quantification of the target compound were performed using the Multiple Reaction Monitoring (MRM) mode. The optimal mass spectrometric parameters for MPQ determination are detailed in Table 2.

**Table 2:** MRM transitions, collision energy, declustering potential and retention times of MPQ.

Compound	MRM [m/z]	Mass transitions	Declustering potential [V]	Collision energy [V]	Retention time [min]
Mepiquat	114 > 98	Quantifier	90	26.1	0.98
	114 > 58	Qualifier	90	25	0.98

### Sample preparation

Sweet potato samples used in this study were sourced from local markets in Giza, Egypt, and stored at − 4 °C until further use. To conduct recovery tests, the samples were spiked with an appropriate concentration of MPQ standard solution. The spiking process was carried out 24 h prior to experimentation to allow sufficient time for the analyte to integrate with the sample matrix, thereby simulating real-world contamination conditions^26^. All experiments were conducted using multiple independently prepared sample portions from different sweet potatoes. Recovery and soil-effect experiments were performed in triplicate (*n* = 3) for each condition.

###  Extraction methods

#### QuEChERS method

A homogenized sample portion weighing 10 ± 0.05 g was measured and placed into a 50 mL polypropylene centrifuge tube. Next, 10 mL of acetonitrile was added to the tube, and the mixture was manually shaken vigorously for one minute. Following this, citrate buffered QuEChERS extraction kits were introduced, and the tube was shaken again for one minute. The mixture was then centrifuged at 4000 rpm for 5 min. After centrifugation, an aliquot of the sample was passed through a 0.45 μm syringe filter, transferred to a vial, and directly analyzed using LC-MS/MS.

#### QuPPe method

For the QuPPe method, 10 ± 0.05 g of homogenized sample was weighed into a 50 mL polypropylene centrifuge tube. Then, 10 mL of methanol containing 1% formic acid was added to the tube. The mixture was shaken for one minute and centrifuged at 4000 rpm for 5 min. Subsequently, an aliquot of the sample was filtered through a 0.45 μm syringe filter, transferred to a vial, and directly analyzed using LC-MS/MS.

### Estimation of soil remaining on sweet potato after washing

The removal of soil from the surface of sweet potatoes during washing was modeled using a first-order exponential decay function. The objective of the model was to estimate the washing time required to reduce the initial soil load to approximately 15% of its original value.

The model assumes an initial soil mass (M₀) of 5 g per sweet potato and simulates the effect of washing under running tap water at a flow rate of 3 L/min with gentle manual scrubbing. The decay constant (k) was calculated based on these predefined boundary conditions, and the model was implemented and solved using Microsoft Excel.

The soil removal process is described by the following equation:

1 $$M(t) = M_{0} *e^{{( - kt)}}$$

Where:

- M(t) is the soil remaining at time t.
- M₀ is the initial soil mass (5 g).
- k is the decay constant.
- t is time in minutes.

**Fig. 1:**
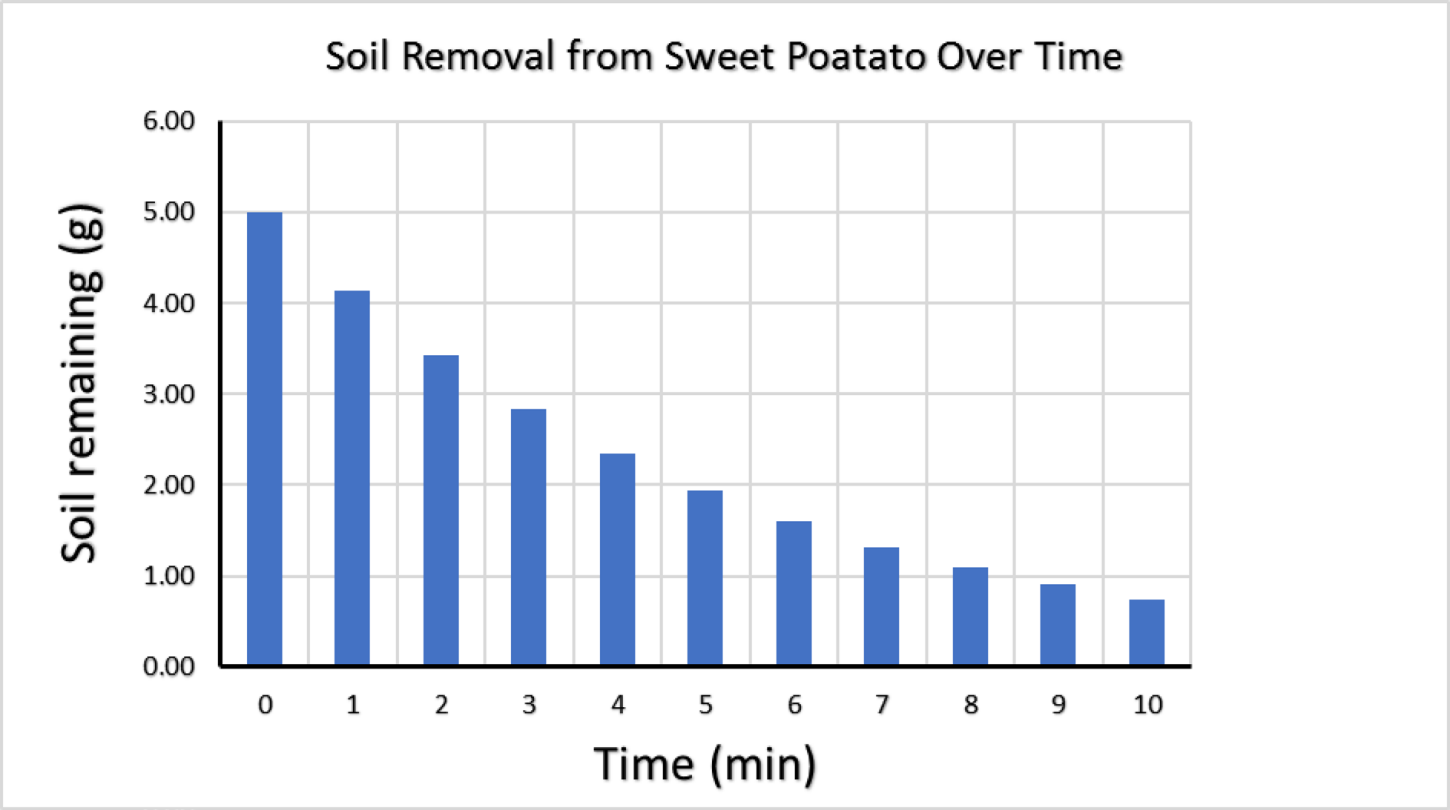
First-order exponential decay model describing soil removal from sweet potato surface during washing.

Table 3 summarizes the input parameters used in the model, and Fig. 1 illustrates the simulated decrease in soil mass as a function of washing time.

**Table 3:** Parameters used to estimate the residual soil amount during washing.

Parameter	Value
Initial soil load	5 g per sweet potato
Water flow rate	3 L/min
Washing duration	10 min
Scrubbing method	Gentle manual scrubbing
Sweet potato surface area	~ 150 cm²

### Method validation

The QuPPe method for the analysis of MPQ was validated in accordance with the European Commission’s SANTE/11,312/2021v2 guidelines. The validation process encompassed the evaluation of key parameters, including specificity, selectivity, trueness (recovery), precision (intra-day repeatability and inter-day reproducibility), calibration linearity, limit of quantification (LOQ), and matrix effect (ME).

Specificity and selectivity were verified by analyzing blank samples. The absence of interfering peaks at the retention time of MPQ confirmed the method’s specificity.

Trueness and precision were assessed through spiked recovery experiments. Blank samples were fortified at two concentration levels (0.02, and 0.1 mg/kg), and six replicates were analyzed for each level. Recovery rates fell within the acceptable range of 70–120%, and the relative standard deviation (RSD) values complied with the validation criteria.

Linearity was evaluated across a concentration range of 0.005–0.5 µg/mL for MPQ in both solvent and matrix-matched extracts. Calibration curves were constructed by plotting concentration (x-axis) against peak area (y-axis) for both matrix-matched and solvent-based standards. Linearity was assessed using the correlation coefficient (R²). The calibration range included concentration levels below the validated LOQ solely for the purpose of assessing linearity. The LOQ was defined as the lowest spiked concentration that met the SANTE/11,312/2021 v2 criteria for trueness (70–120%) and precision (RSD < 20%), which in this study corresponded to 0.02 mg/kg.

Matrix effects, which can cause signal suppression or enhancement, were observed in food matrices by comparing calibration solvent injections vs. matrix matched calibration injections.

The LOQ was determined as the lowest validated spiked concentration that met the trueness and precision requirements, with recovery rates within the 70–120% range and RSD < 20%.

Accuracy and precision were further evaluated through spiked recovery experiments. Intra-day precision (repeatability) was assessed by analyzing fortified samples on the same day, while inter-day precision (reproducibility) was determined by analyzing samples over three consecutive days. Precision was evaluated by calculating the RSD values.

## Real samples collection

Thirty independent sweet potato samples were collected from different vendors and locations within Giza markets to ensure sample variability and representativeness. For practical analysis, the samples were divided into two batches: 15 samples were collected and analyzed on the first day, and the remaining 15 samples were collected and analyzed on the second day. This approach ensured efficient processing and minimized potential delays in sample analysis.

All samples were transported to the laboratory under controlled conditions to maintain their integrity. Upon arrival, the samples were prepared and analyzed using the validated QuPPe method for MPQ residue detection.

## Result and discussion

### Residual soil effect

Initially the aim of this group was to use the QuEChERS method for the determination of MPQ in sweet potatoes. The original aim was to test the spiking recovery, if it ranged from 70 to 120% then it passes, if it ranged from 30 to 69 or 121–140% then a compensation would have been necessary, pending that the RSD% is < 20%, per the SANTE/11,312/2021v2 guidelines. The first recovery studies (0.05 mg/kg, *n* = 3) were done on a blank sweet potato sample tested by the QuEChERS method to make sure that it has no MPQ residue. Furthermore, the sample was washed with running water for 15 min to eliminate the possibility of having residual MPQ with a < LOQ value that may interfere with the recovery test. The mean recovery for that test was 72%, a borderline accepted result for the EU SANTE guidelines criteria (70–120%). This recovery is unsettling since it doesn’t take into consideration the residual soil effect that was previously observed for some quaternary ammonium salts. In a report published by the EURL-SRM, it was reported that the recovery of diquat (DQ), and paraquat (PQ) (another quaternary ammonium salts pesticides) in potatoes are highly affected by the presence of soil even when using internal standards^28^. Furthermore, Juhler et al. reported that chlormequate was highly adsorbed on soil with a very low desorption rate even under favored conditions^29^.

According to the Codex Alimentarius Commission CAC/GL 41-1993 guidelines, it is a common practice to remove loose soil and debris from tubers using washing under running water and soft brush before commuting. However, it is practically impossible to remove all the soil using a brush only without removing parts of the sweet potato itself which may compromise the analysis step. Washing with running water may also affect the concentration of the incurred pesticides^30^. Using a first-order exponential decay model according to Eq. (1), it was practically impossible to remove all the soil using only washing and scrubbing; even after 10 min, a residual amount of soil remained, as shown in Fig. 1.

To test if washing for this time will affect the concentration of MPQ in a sample, a positive potato sample was taken and cut in half, one was washed and the other wasn’t washed. The washed part showed a decrease from 0.1 mg/kg to 0.072 mg/kg when analyzed using the QuPPe method. Therefore, washing is not a viable option for this compound. Also spiking reproducibility was done on different blank dry brushed samples, a fluctuation of recovery was observed where the recovery ranged from 30 to 65%.

This lead us with the conclusion that MPQ, same as other quaternary ammonium salt pesticides, can be affected by soil. Further verification was done using, a systematic study on the effect of the presence of residual soil in the sweet potatoes samples and the recovery of MPQ using QuEChERS and QuPPe methods, taking into consideration the nature of the Egyptian agriculture land of the sandy loam and clay loam^31^.

**Fig. 2:**
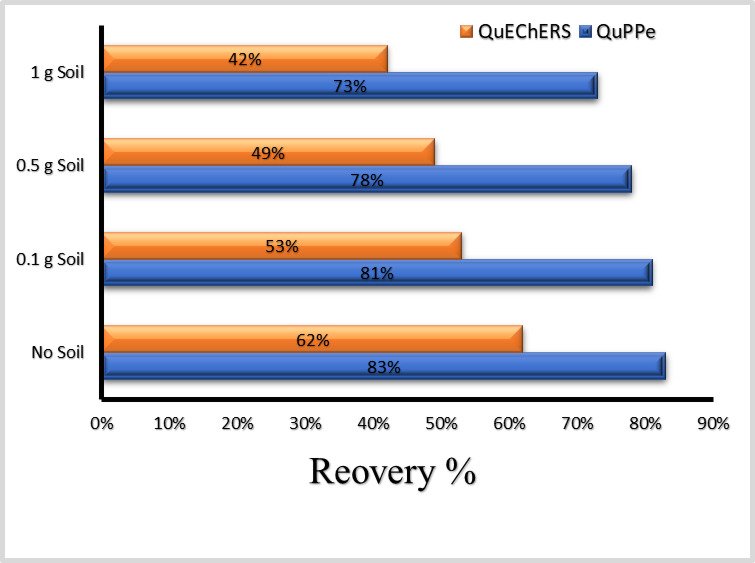
Effect of Soil Residue on MPQ Recovery in Sweet Potatoes Using QuEChERS and QuPPe Methods.

Figure 2 illustrates the recovery of MPQ (0.05 mg/kg, *n* = 3) in sweet potatoes while systematically adding incremental portions of two soil types (sandy loam and clay loam) to the sample (0.1, 0.5, 1 g added to the sample portion). Each soil level and soil type was tested in triplicate using independently prepared samples. The results indicate that the QuPPe method was significantly less affected by the presence of soil compared to the QuEChERS method.

Additionally, the type of soil had a notable impact on the recovery rates. Sandy loam had a less adverse effect than clay loam, even when using the QuEChERS method. The recoveries in sandy loam were more than half the magnitude of those in clay loam. This observation underscores the importance of considering soil type in the analysis of pesticide residues in agricultural products.

Given these findings, the QuPPe method was selected for the monitoring of MPQ in sweet potatoes in our case due to its superior performance in the presence of soil residues. This choice aligns with the need for accurate and reliable pesticide residue analysis, particularly in regions with varying soil compositions like Egypt.

### Method validation

The validation results are based on multiple independently prepared replicates and demonstrate the reliability and robustness of the method under routine laboratory conditions. The validation of the developed method for analyzing MPQ in sweet potatoes was performed in compliance with the European Commission guidelines (SANTE/11312/2021 v2). The validation involved the assessment of key performance parameters, including specificity, selectivity, trueness (recovery), precision, linearity, matrix effects, and limit of quantification, to ensure the method’s suitability for quantitative MPQ analysis in sweet potatoes.

Specificity and selectivity were confirmed through analyzing blank sweet potatoes samples, where no interfering peaks were detected at the MPQ retention time. In order to assess accuracy and precision, six replicates of spiked samples were analyzed at two concentrations of 0.02 mg/kg and 0.1 mg/kg (taking into consideration the dilution factor). The spiked samples recovery ranged from 83% to 117%, with relative standard deviation (RSD) values between 1.19% and 1.38% (Table 4), demonstrating the method robustness and applicability for routine analysis of MPQ in sweet potatoes. The retention time stability was confirmed by then injection of 10 0.1 mg/kg MPQ calibration standard using the developed method, a variation in retention time with ± 0.1 was found. A five level calibration (0.005, 0.01, 0.05, 0.1, and 0.5 µg/mL) was used to examine the linearity of the method. The Method showed a correlation coefficient (r^2^) greater than 0.999, confirming the method linearity across the studied concentrations.

**Table 4:** Accuracy and precision of MPQ analysis in sweet potatoes.

Concentration (mg/kg)	Recovery (%)	RSD (%)	Average Recovery (%)	Average RSD (%)
0.02	105	1.28	**102.2**	**1.25**
0.02	102	1.28		
0.02	117	1.19		
0.02	86	1.21		
0.02	116	1.27		
0.02	87	1.29		
0.1	84	1.28	**85.5**	**1.31**
0.1	84	1.32		
0.1	89	1.27		
0.1	83	1.29		
0.1	86	1.38		

The LOQ of the method was set to 0.02 mg/kg, as this was the lowest concentration level that fulfilled the SANTE/11,312/2021 v2 requirements for accuracy and precision, although linearity was evaluated down to lower concentration levels.

#### Matrix effect

The sample components other than the analytes being observed is known as the matrix. The influence and interference of the sample matrix and the analyte being investigated is known as matrix effect (ME). In LC-MS/MS, the analyte usually encounter signal suppression due to the competition between the analyte and the matrix during sample ionization in the ion source^32^. Two single point level matrix matched calibration of MPQ (at 0.01 and 0.05 µg/mL) was prepared in sweet potatoes extract to evaluate ME. The signal values were − 10% and − 18% suppression when compared with the solvent calibration. This value is within the range of the ± 20%. Additionally, to compensate for the observed ME, matrix matched calibration was used for quantification.

### Real samples survey

**Fig. 3:**
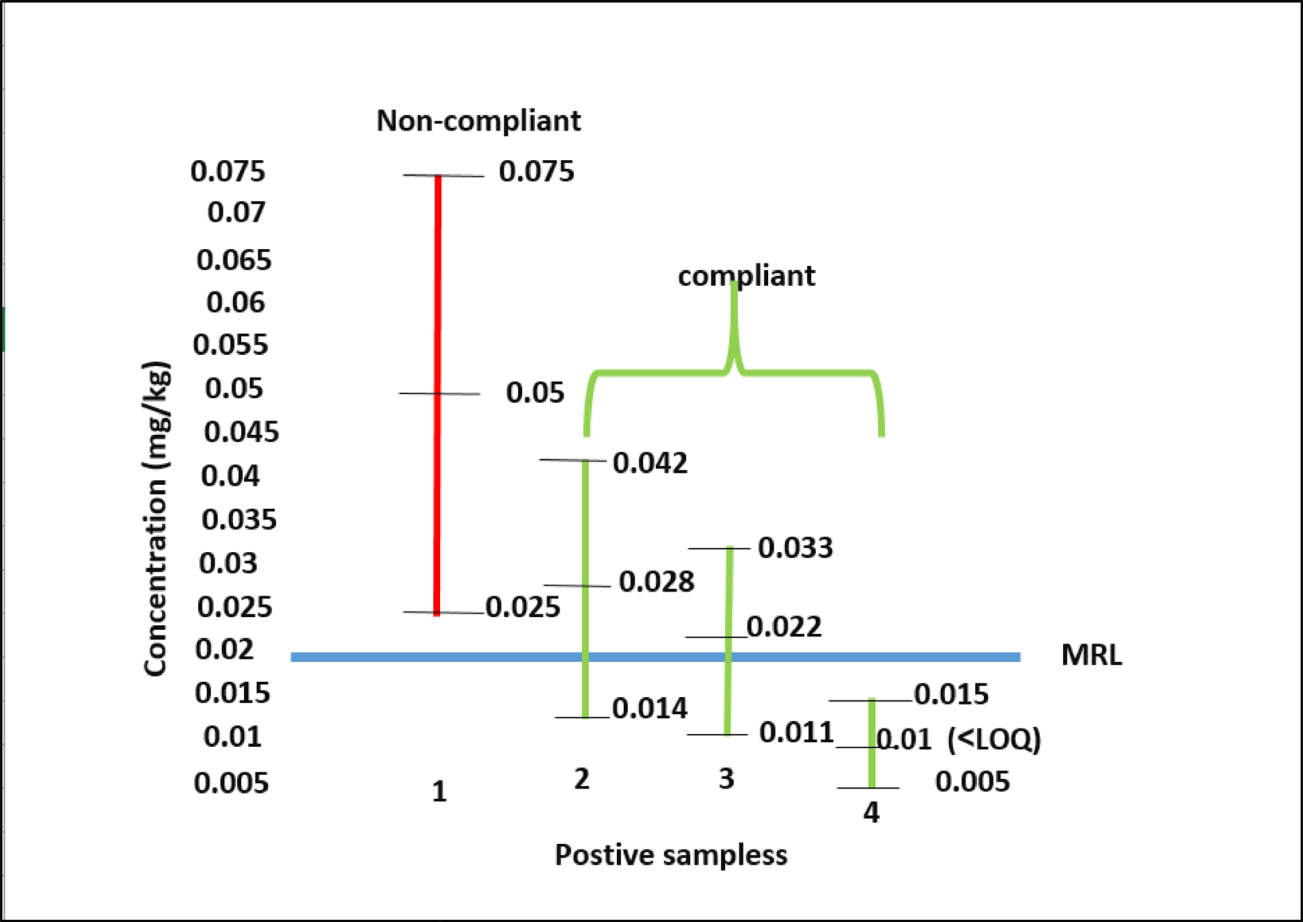
Concentration of MPQ residues in detected sweet potato samples relative to EU MRL standards.

The method applicability was investigated through the analysis of thirty real sweet potatoes samples collected from local markets in Giza, Egypt. As shown in Fig. 3, out of the thirty samples, only four samples were observed to have MPQ residues. One sample had a residue l, another had 0.022 mg/kg, the third 0.028 mg/kg, and the fourth 0.05 mg/kg. The MPQ MRL in the EU is 0.02 mg/kg. Hence, practically, three out of the four positive samples are within the acceptance limit (one under the MRL, and two accepted taking into consideration the uncertainty value as stated in the SANTE/11312/2021v2 guidelines). However, the sample size is too small to accurately determine the MPQ contamination situation in Egypt.

## Conclusion

Mepiquat is an analytically challenging pesticide that needs many precautions in its analysis in sweet potato samples. Two main factors were detrimental in this study; (a) the extraction solvent polarity, (b) the residual soil in the sample before its commuting. Hence, it is recommended to use the QuPPe “dilute and shoot” method, in combination with a careful yet thorough brushing of the soil and debris particles from the sweet potatoes. Mepiquat was found in 4/30 Egyptian sweet potatoes samples, with only one exceeding the limit. However, a bigger survey should be done to further understand the mepiquat contamination in the Egyptian sweet potatoes.

## Data Availability

The datasets generated and analysed during this study, including raw LC–MS/MS files, and recovery validation results, are available from the corresponding author upon request.
